# ZBTB7A suppresses glioblastoma tumorigenesis through the transcriptional repression of EPB41L5

**DOI:** 10.1038/s12276-022-00908-8

**Published:** 2023-01-04

**Authors:** Ji-Hoon Jeong, Seung-Ho Park, Hyunhee Kim, Hae Yun Nam, Sung-Hak Kim, Minseok Jeong, Min-Jeong Kong, Jihyun Son, Ji-Eun Jeong, Ji-Hye Song, Seong Who Kim, Kyung-Chul Choi

**Affiliations:** 1 Department of Biomedical Sciences, AAMIST, Asan Medical Center, University of Ulsan College of Medicine, Seoul, Korea; 2 Department of Biochemistry and Molecular Biology, AMIST, Asan Medical Center, University of Ulsan College of Medicine, Seoul, Korea; 3 Department of Animal Science, Chonnam National University, Gwangju, Korea; 4 Korea Food Research Institute, Wanju-gun, 55365 Korea

**Keywords:** Tumour biomarkers, Gene regulation

## Abstract

Glioblastoma multiforme (GBM), the most aggressive and malignant glioma, has a poor prognosis. Although patients with GBM are treated with surgery, chemotherapy, and radiation therapy, GBM is highly resistant to treatment, making it difficult and expensive to treat. In this study, we analyzed the Gene Expression Profiling Interactive Analysis dataset, the Cancer Genome Atlas dataset, and Gene Expression Omnibus array data. ZBTB7A (also called FBI1/POKEMON/LRF) was found to be highly expressed in low-grade glioma but significantly downregulated in patients with GBM. ZBTB7A is a transcription factor that plays an important role in many developmental stages, including cell proliferation. The activation of epithelial-mesenchymal transition (EMT) is a key process in cancer progression and metastasis. Erythrocyte membrane protein band 4.1 like 5 (EPB41L5) is an essential protein for EMT progression and metastasis in various types of cancer. We found that ZBTB7A depletion in U87 cells induced GBM progression and metastasis. Based on RNA sequencing data, ZBTB7A directly binds to the promoter of the EPB41L5 gene, reducing its expression and inhibiting GBM progression. We demonstrated that ZBTB7A dramatically inhibits GBM tumor growth through transcriptional repression of EPB41L5. Thus, both ZBTB7A and EPB41L5 may be potential biomarkers and novel therapeutic targets for GBM treatment. Overall, we discovered the role of a novel tumor suppressor that directly inhibits GBM progression (ZBTB7A) and identified EPB41L5 as a therapeutic target protein for patients with GBM.

## Brain tumors: Regulatory protein suppresses tumor growth

The identification of a key tumor-suppressing protein could provide biomarkers and treatments for an aggressive and fast-growing form of brain and spinal cord cancer. Glioblastoma multiforme (GBM) is resistant to many forms of treatment, resulting in poor prognosis. By analyzing gene expression databases, Kyung-Chul Choi and Seong Who Kim at the University of Ulsan College of Medicine in Seoul, South Korea, and co-workers found that expression of a regulatory protein, the transcription factor ZBTB7A, is significantly reduced in patients with aggressive GBM. In experiments on mice, the researchers found that ZBTB7A inhibits expression of the *EPB41L5* gene, which codes for a protein which promotes tumor growth and metastasis. Both the ZBTB7A and EPB41L5 proteins could provide useful biomarkers for GBM prognosis, and offer therapeutic targets for the disease.

## Introduction

The most common and fatal primary tumor is glioblastoma multiforme (GBM); it is also the highest grade (grade IV) human brain tumor^1–4^. Despite surgical interventions such as chemotherapy and radiation therapy, GBM is now resistant to all treatments and is fatal with a 14–16 month survival rate^5–9^. The development of resistance to standard treatment regimens is a major cause of poor survival^10–12^, and because GBM is highly invasive and aggressive, patient prognosis is poor despite various drug treatment options^13,14^. According to cohort molecular profiling studies, genetic abnormalities frequently occur in specific genes, such as PTEN, TP53, and IDH1, in patients with GBM^15^. Thus, prognosis and treatment response prediction are difficult for patients with GBM^16^, suggesting that there are other factors contributing to aberrant gene expression because of the molecular heterogeneity of tumor tissue. Therefore, the development of novel therapeutic strategies to increase the survival rate of GBM requires the analysis of gene, protein, and molecular mechanisms through gene profiling, and it is critical to identify key GBM diagnostic markers.

ZBTB7A, also known as FBI1, POKEMON, or LRF, is a member of the POK family and a pleiotropic transcription factor containing zinc fingers and protein 7A^17–19^. Transcription factors of the POK family act as transcription activators or repressors by specifically binding to DNA elements located on the DNA-binding domain of target genes^20^ and can also repress transcription by recruiting corepressor complexes^21^. In addition, overexpression of ZBTB7A is associated with tumorigenesis and metastasis in various human cancers^22^. For example, ZBTB7A inhibits transcription by directly binding to GLUT3, PFKP, and PKM, all of which are important factors in glycolysis, as well as the promoter of the androgen receptor gene in prostate cancer^20,23–26^. In addition, ZBTB7A acts as a tumor suppressor; the loss of ZBTB7A in a PTEN-deficient environment promotes tumor growth in mouse prostate cancer^24^. However, the mechanisms of gene regulation by ZBTB7A in GBM cells remain unknown.

EPB41L5 (erythrocyte membrane protein band 4.1 Like 5) is a mesenchymal-specific protein induced during EMT (epithelial-mesenchymal transition) that promotes the disruption of cell‒cell adhesion kinetics^27^. Overexpression of EPB41L5 in epithelial cells causes ZO-1 disorganization, as it destabilizes E-cadherin and affects the tightness of cell junctions^28,29^. EPB41L5 is highly expressed in gastric, kidney, and breast cancers and promotes invasion, metastasis, and EMT^30–33^. It is also considered a key factor in the metastasis of various types of cancer by promoting EMT, proliferation, migration, and invasion in esophageal squamous cell carcinoma^27^. However, the role and function of EPB41L5 in GBM remain unclear.

In this study, we confirmed that the expression of ZBTB7A was very low in GBM through the analysis of The Cancer Genome Atlas (TCGA) dataset, and the decreased expression of ZBTB7A was correlated with lower survival rates of GBM. Interestingly, ZBTB7A is associated with cell migration, and upregulation of ZBTB7A significantly suppressed migration, invasion, and proliferation in GBM. ZBTB7A negatively regulates GBM tumor progression by directly binding to the promoter of *EPB41L5*, an important gene involved in cell mobility, and repressing its transcription. Our study revealed a novel tumor suppressor role for ZBTB7A, which directly inhibits GBM tumorigenesis. In addition, we discovered that EPB41L5 is a key marker of GBM tumorigenesis and is regulated by the transcriptional repression of ZBTB7A. Therefore, we suggest that the coregulation of ZBTB7A and EPB41L5 is an essential therapeutic strategy for treating GBM.

## Materials and methods

### Cells, plasmids, and reagents

Human A172, LN18, LN229, U118, U343, U373, and U87 cells were cultured in Dulbecco’s modified Eagle’s medium (DMEM; Biowest, L0103) supplemented with 10% fetal bovine serum (FBS; Biowest, S1480) and 1% antibiotics (HyClone, SV30010). All cells were cultured at 37 °C in a 5% CO_2_ incubator. Effectene transfection reagent was purchased from QIAGEN (301427). The Myc-tagged ZBTB7A constructs were subcloned into the pSG5 vector. The pCDH-CMV-MCS-EF1-Puro overexpression vector was purchased from System Biosciences (CD510B-1). The shRNAs were obtained from Mission-shRNA (Sigma‒Aldrich). Stably overexpressed LN229, U343, and knockdown U87 cells were cultured under puromycin selection (1.5 μg/mL). The shRNA clone IDs are listed in Supplementary Table 1.

### In vitro migration, invasion, colony formation, and proliferation assays

After stable overexpression LN229 and U343 and knockdown U87 cells reached ~70% confluence, the cells were scratched with a 200 µL pipette tip, washed with DPBS, and incubated at 37 °C. Wound healing was observed for 12 h and 24 h at the scratched line, and the area around the scratched line was photographed. The invasion assay was performed using an 8.0 μm pore polycarbonate membrane insert (Corning, 353097). Transwell inserts coated with Matrigel (Corning, 354234) were used, and 2 × 10^4^ cells/well were plated in the upper chamber. The lower chamber was filled with 600 μL of serum-free medium. After incubation for 48 h, cells infiltrated with 100% methanol were fixed. After staining with 1% crystal violet (Sigma‒Aldrich, V5265), the infiltrated cells were observed under a microscope. For the colony formation assay, 1 × 10^3^ cells were plated in 6-well plates. After culturing the cells at 37 °C for 2 weeks, colonies formed on each of the three plates were measured. Cell proliferation was assessed according to the manufacturer’s protocol (Invitrogen, C35011) using the CyQuant Direct Cell Proliferation Assay Kit. All experiments were performed in triplicate, and cell proliferation was determined using a VICTOR^TM^ X3 Multilabel Plate Reader (PerkinElmer).

### Western blot analysis

Cells were harvested for Western blotting by scraping and washing with cold phosphate-buffered saline (PBS). The cells were lysed with lysis buffer for 30 min on ice. Lysates were centrifuged at 13,000 *rpm* for 20 min at 4 °C, and the protein concentration of the supernatant was measured using Pierce 660 nm Protein Assay Reagent (Thermo, 22660). Total cell lysate proteins were separated using sodium dodecyl sulfate‒polyacrylamide gel electrophoresis. The separated proteins were transferred to nitrocellulose membranes. The transferred membranes were blocked by incubation for 1.5 h in 5% w/v nonfat Difco skim milk (BD Biosciences, 232100) blocking buffer. The primary antibodies were incubated together for 3 h or overnight at 4 °C. After washing, the secondary antibody was incubated for 1.5 h. The results were visualized by the developer after Western blot analysis. The antibodies used were anti-ZBTB7A (Abcam, ab70208), anti-EPB41L5 (Thermo, PA5-58009), anti-β-actin (Sigma‒Aldrich, A5441), anti-Myc (MBL, M192-3), anti-N-cadherin (sc-59987), anti-β-catenin (sc-7963), anti-vimentin (sc-32322) (Santa Cruz Biotechnology), and anti-Snail (Cell Signaling, 3879 S). All secondary antibodies were purchased from Thermo (31430 and 31460).

### RNA extraction and quantitative polymerase chain reaction

Total RNA was isolated using an AccuPrep Universal RNA Extraction Kit (BIONEER, K-3141) according to the manufacturer’s instructions. Total RNA was converted to cDNA by cDNA synthesis using the PrimeScript RT Reagent Kit (Takara, 2680 A). For real-time quantitative polymerase chain reaction (qPCR), the primer sequences used for the *ZBTB7A*, *EPB41L5*, *APOE*, *MDK*, *MMP9*, *IL27RA*, *SEMA3A*, *FGF7*, *IL1B*, *PAK3*, *ADAM18*, *SERPINF1*, *TGFB2*, *HYAL1*, *THBS1*, *IL33*, *ITGA2*, *ACVRL1*, *CXCL8*, *CDH2* (N-cadherin), *CTNNB1* (β-catenin), *VIM* (vimentin), and *GAPDH* genes are listed in Supplementary Table 1. qPCR was performed using a real-time PCR kit (ELPIS Bio, EBT-1801), and all samples were normalized using the ΔΔCt method. All expression values are expressed as the fold change, and the reaction was repeated three times.

### Luciferase reporter gene assay

The human *EPB41L5* gene promoter was prepared and purified by PCR amplification of human genomic DNA from HEK293T cells for a specific region (−900 to 100 nucleotides) of the *EPB41L5* gene. It was cloned into the pGL4.21 (*luc2P*/Puro) vector (Promega, E6761) using *KpnI* and *XhoI* restriction enzymes. Luciferase analysis of the EPB41L5 reporter gene and *β-galactosidase* gene was transiently transfected with 100 ng and 400 ng per well, respectively. After incubation for 24 h, the luciferase assay system (Promega, E1501) was used according to the manufacturer’s instructions. The colorimetric substrate ortho-nitrophenyl-galactoside was used to measure β-galactosidase activity at 405 nm absorbance. Normalized activity was expressed as luciferase activity/β-galactosidase activity (fold change).

### Chromatin immunoprecipitation assay

Chromatin immunoprecipitation (ChIP) assays were performed on U343 cells, stably overexpressed U343 cells and ZBTB7A-knockdown U87 cells using the Pierce Magnetic ChIP Kit (Thermo, 26157). Nuclear lysates were precipitated with anti-ZBTB7A, anti-Myc, normal rabbit (sc-2027), and mouse (sc-2025) IgG (Santa Cruz Biotechnology) overnight at 4 °C with A/G magnetic beads. DNA obtained following the manufacturer’s instructions was analyzed using qPCR. The ChIP primers (−833/−649, −314/−177, −202/−58, and −130/−56) used for qPCR are shown in Supplementary Table 1.

### Animal studies

At the University of Ulsan College of Medicine, all mouse experiments were performed under an approved protocol (2020-12-353). This study was conducted in accordance with the guidelines of the International Animal Care and Use Committee. Briefly, stably overexpressed U343 (5 × 10^5^) cells were collected and injected subcutaneously into the flanks of female BALB/c-nu mice. After 5 weeks, all mice were sacrificed. Tumor volume was measured every 2–3 days and calculated using the formula V = (L × W^2^)/2 (L; longer, W; width). Subsequently, the tumors were excised, weighed, and photographed. The cells used for the orthotopic xenograft mouse model were U87 (1 × 10^6^) cells with stable knockdown of ZBTB7A, and these cells were injected into the right hemisphere. After 4 weeks, all mice were sacrificed. For all animal experiments, mice were randomly allocated to each experimental group.

### Immunohistochemistry analysis

The immunohistochemistry (IHC) detection kit was purchased from Abcam (ab64264). Experiments were performed according to the manufacturer’s instructions. Anti-ZBTB7A, anti-EPB41L5, and Ki67 (Abcam, ab16667) were diluted 1:300. Images were analyzed using ImageJ software (Java 1.8.0_112, NIH) and an IHC profiler (special plug-in).

### Analysis of datasets for human samples and cell lines

Various data on differential expression between tumor/normal tissues were obtained from the Gene Expression Profiling Interactive Analysis (GEPIA) database (http://gepia.cancer-pku.cn/index.html). Data on human TCGA-GBM (*n* = 538) and TCGA-GBMLGG (*n* = 667), including overall survival and correlation data, were obtained from the GlioVis database (http://gliovis.bioinfo.cnio.es/). Affymetrix Human Genome U133A Array (GPL96) CEL files were obtained from the Freije dataset from Gene Expression Omnibus (GEO) with accession number GSE4412. The Affymetrix ID used was 213299_at. Human glioma cancer cell line (*n* = 59) expression data were obtained from the Cancer Cell Line Encyclopedia (CCLE) database (https://sites.broadinstitute.org/ccle/). The target gene prediction data for ZBTB7A were obtained from the ChIP-Atlas database (https://chip-atlas.org/). The predicted ZBTB7A binding motif sequence and the promoter sequence of *EPB41L5* were analyzed using JASPAR (https://jaspar.genereg.net/) and the Eukaryotic Promoter Database (EPD; https://epd.epfl.ch//index.php).

### mRNA sequencing analysis

Total RNA was isolated using TRIzol reagent (Invitrogen, 15596018), and RNA quality and quantification were performed using an Agilent 2100 bioanalyzer (Agilent Technologies) and an ND-2000 Spectrophotometer (Thermo). Library preparation was performed using the NEBNext Ultra II Directional RNA-Seq Kit (NEW ENGLAND BioLabs, E7760L). Using a Poly(A) RNA Selection Kit, mRNA was extracted (LEXOGEN, 157.96) and used according to the manufacturer’s instructions for cDNA synthesis and shearing. Indexing was performed using Illumina indices 1–12 and was enriched by PCR. The mean fragment sizes were evaluated, and libraries were checked using a TapeStation HS D1000 Screen Tape (Agilent Technologies). Quantification was performed using the StepOne Real-Time PCR System (Life Technologies), and high-throughput sequencing was performed using NovaSeq 6000 (Illumina). FastQC was used to control the quality of the raw sequencing data, and adapters and low-quality reads (<Q20) were removed using FASTX_Trimmer and BBMap. TopHat was used to map the trimmed reads to the reference genome, and fragments per kilobase per million read (FPKM) values were used to estimate gene expression levels. FPKM values were normalized using EdgeR in R (R Development Core Team). Data mining and graphical visualization were performed using Microsoft Excel (Office 365), GSEA (GSEA_4.1.0, Broad Institute), and ExDEGA (Ebiogen).

### Statistical analysis

Statistical significance was determined using Student’s *t* test or one-way ANOVA. Prism (Prism 8.0.1, GraphPad) and Excel (Microsoft) were used. All values are reported as the mean ± SD. A *p* value < 0.05 was considered to indicate significance (*p < 0.05, **p < 0.01, ***p < 0.001).

## Results

### ZBTB7A expression was decreased in GBM cells and patients

**Fig. 1:**
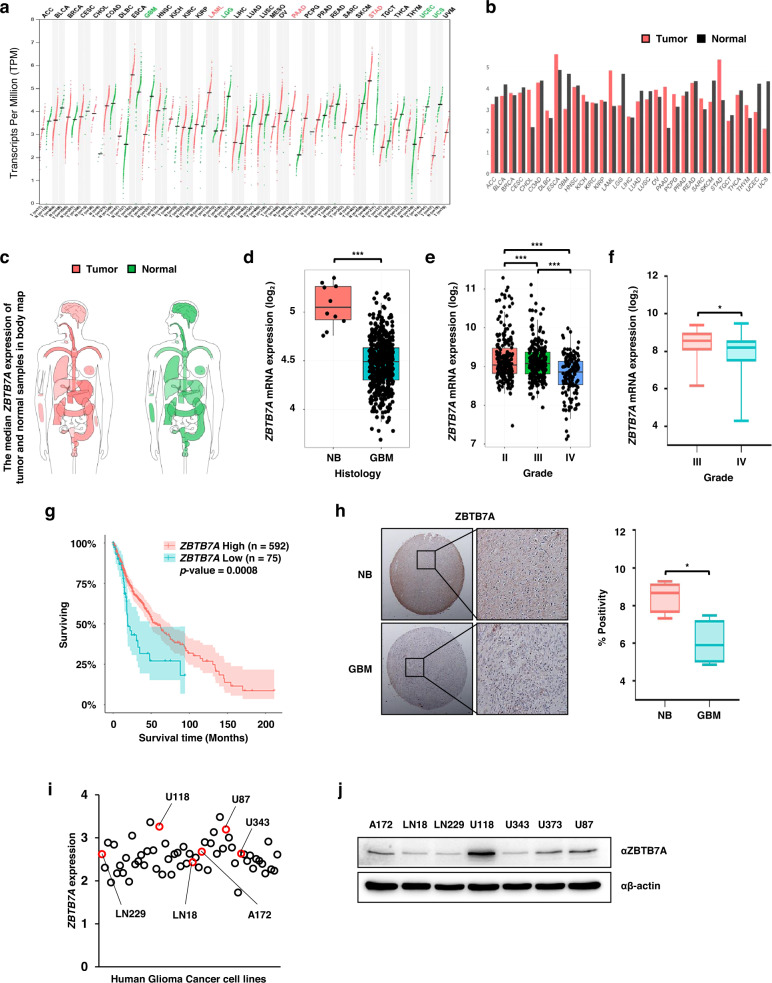
ZBTB7A was decreased in GBM cells and patients. **a**, **b** *ZBTB7A* expression in cancers. *ZBTB7A* expression profiles across tumor samples compared to NB samples for 33 TCGA tumor types using GEPIA. The mean log_2_(TPM + 1) was used as the signature score. Each point on the dot plot represents the sample, and the height of the bars in the bar plot represents the median expression of a specific tumor type or NB. **c** Median *ZBTB7A* expression in tumor and NB samples in the interactive bodymap (GEPIA). **d** mRNA expression of human *ZBTB7A* in NB and GBM samples from TCGA-GBM (GlioVis). NB, *n* = 10; GBM, *n* = 528. NB – GBM, *p* = 3.1E−13. **e** mRNA expression levels of *ZBTB7A* in gliomas grades II, III, and IV in TCGA-GBMLGG (GlioVis). Grade II, *n* = 226; III, *n* = 224; IV, *n* = 150. II–III, *p* = 6.4E−01; II–IV, *p* = 7.8E−12; III–IV, *p* = 4.4E−09. **f** mRNA *ZBTB7A* expression analysis using the Freije dataset (GEO; GSE4412). Grade III, *n* = 26; IV, *n* = 59. **g** Kaplan–Meier curves representing the survival of patients with low versus high expression of *ZBTB7A* in TCGA-GBMLGG (GlioVis). **h** IHC analysis of TMAs in human NB and GBM samples using anti-ZBTB7A antibody. NB, *n* = 4; GBM, *n* = 4. **i** Analysis of the *ZBTB7A* expression profiles in glioma cell lines using CCLE. **j** ZBTB7A protein expression levels in GBM cells. Scale bars: 50 μm. *p < 0.05.

We investigated the RNA expression of *ZBTB7A* in GBM patients using various databases. Profiling of various tumor samples and normal brain (NB) tissues from the TCGA and the Genotype-Tissue Expression (GTEx) projects in GEPIA confirmed that *ZBTB7A* expression was lower in GBM and lower-grade glioma tissues than in NB tissues (Fig. 1a–c). Analysis of TCGA-GBM and TCGA-GBMLGG datasets using the GlioVis database revealed that *ZBTB7A* expression was significantly decreased in GBM, and it was confirmed that the expression of *ZBTB7A* was decreased in high-grade tumors (Fig. 1d, e). In addition, GEO profiling revealed that the expression of *ZBTB7A* in the Freije dataset (GSE4412) was decreased in GBM, as was observed in the TCGA-GBMLGG dataset (Fig. 1f). Furthermore, the prognosis of patients with GBM was poor in the GBM patients with *ZBTB7A* downregulation (Fig. 1g) according to Kaplan–Meier survival analysis based on the TCGA-GBMLGG dataset. To determine whether the expression of ZBTB7A was decreased in GBM tissues compared to NB tissues based on the results of the analysis of various databases, we performed IHC using tissue microarrays (TMAs). The expression of ZBTB7A was significantly decreased in GBM tissues (Fig. 1h). The expression of *ZBTB7A* was confirmed using the CCLE database before the in vitro experiment, and *ZBTB7A* expression was found to be relatively low in LN229, LN18, A172, and U343 cells and relatively high in U118 and U87 cells (Fig. 1i). In addition, when the CCLE database was used to analyze ZBTB7A protein expression in the GBM cell line, nearly identical results were obtained (Fig. 1j). Based on these results, the expression of ZBTB7A is decreased in both GBM cell lines and GBM patients, and decreased expression of ZBTB7A at the mRNA and protein levels indicates a poorer prognosis.

### ZBTB7A knockdown promotes GBM tumorigenesis

**Fig. 2:**
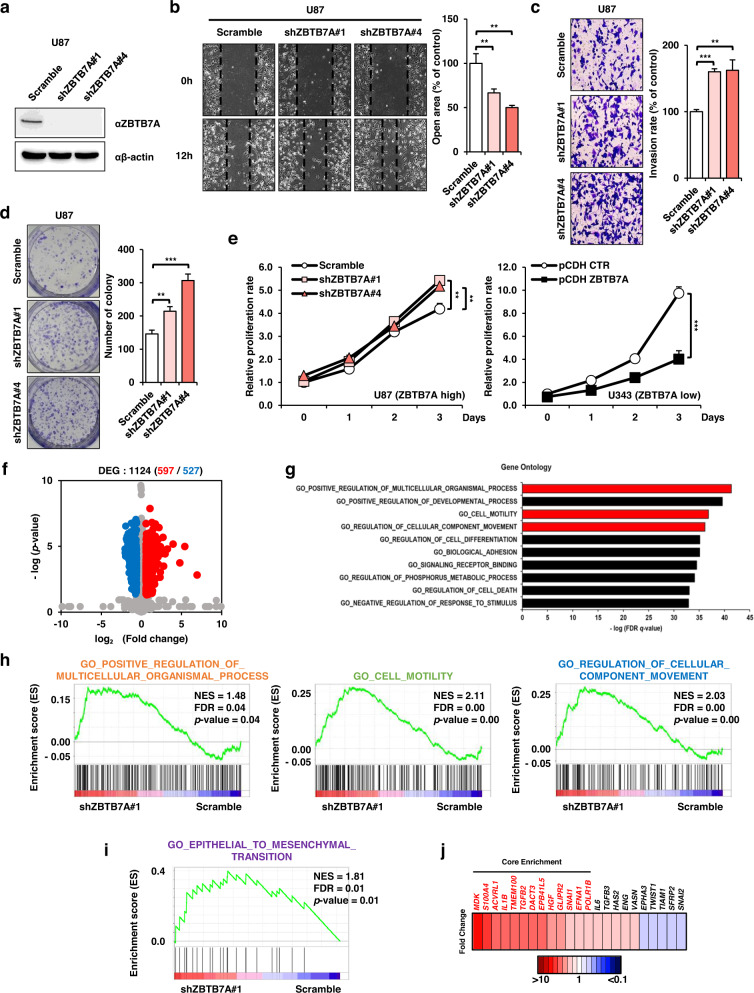
*EPB41L5* is the target gene of ZBTB7A in GBM cells. **a** Western blot analysis of ZBTB7A in shZBTB7A U87 cells. **b** Migration assay of shZBTB7A U87 cells after 12 h. **c** Transwell invasion assay of shZBTB7A U87 cells after 48 h. **d** Effect of ZBTB7A knockdown on colony formation in shZBTB7A U87 cells. **e** Cell proliferation assay was performed after shZBTB7A U87 and pCDH ZBTB7A U343 cells for 3 d. **f** Differentially expressed gene analysis of mRNA-seq of shZBTB7A U87 cells (red dots; upregulated genes, blue dots; downregulated genes, FC ± 1.5 and *p value* < 0.05). **g** Gene Ontology enrichment analysis for up- and downregulated genes scramble versus shZBTB7A. **h**, **i** GSEA revealed differences in the expression of genes involved in cell migration and EMT. **j** EMT-related core enrichment gene fold change value. NES (normalized enrichment score), FDR (fold discovery rate), **p < 0.01, ***p < 0.001.

We examined the tumorigenicity of U87 cells, a cell line with relatively high expression of ZBTB7A, after transfection with ZBTB7A shRNA or control shRNA lentiviral plasmid (Supplementary Fig. 1a; Control shRNA and shZBTB7A#1–6) to determine the probable role of ZBTB7A in GBM development. In addition, we overexpressed ZBTB7A in LN229 and U343 cells, which have relatively low ZBTB7A expression. To investigate the tumorigenicity of ZBTB7A-depleted U87 cells, we assessed the protein and mRNA expression levels of ZBTB7A (Fig. 2a, Supplementary Fig. 1b). When the expression of ZBTB7A was knocked down, cell migration was significantly increased in ZBTB7A-knockdown U87 cells (Fig. 2b), and tumor invasion and colony formation were also increased in ZBTB7A-knockdown U87 cells compared with control U87 cells (Fig. 2c, d). In addition, we performed a proliferation assay. Cell proliferation was significantly promoted in ZBTB7A-knockdown U87 cells and conversely inhibited in ZBTB7A-overexpressing U343 cells (Fig. 2e). Therefore, our results suggest that ZBTB7A acts as a tumor suppressor in GBM.

To gain insight into the mechanism of action of ZBTB7A, we performed mRNA sequencing analysis. Of the 1124 genes, 597 were upregulated and 527 were downregulated (DEG analysis) (Fig. 2f). We also performed gene ontology analysis of 1124 upregulated and downregulated genes. These results showed that multicellular organisms were enriched for genes involved in processes, cell motility, and the movement of cellular components (Fig. 2g). GSEA revealed that all genes involved in the three processes were more enriched in the GBM group than in the control group when ZBTB7A was knocked down (Fig. 2h, Supplementary Table 2), suggesting that ZBTB7A is generally involved in biological processes related to cell motility. Thus, we further confirmed whether the knockdown of ZBTB7A plays an important role in the EMT process due to increased cellular motility. When ZBTB7A was knocked down in GBM cells, the EMT process was induced, and the related core genes were enriched (Fig. 2i, j), indicating that ZBTB7A knockdown activates GBM growth-related genes to facilitate the development and progression of GBM.

### EPB41L5 is the target gene of ZBTB7A in GBM cells

**Fig. 3:**
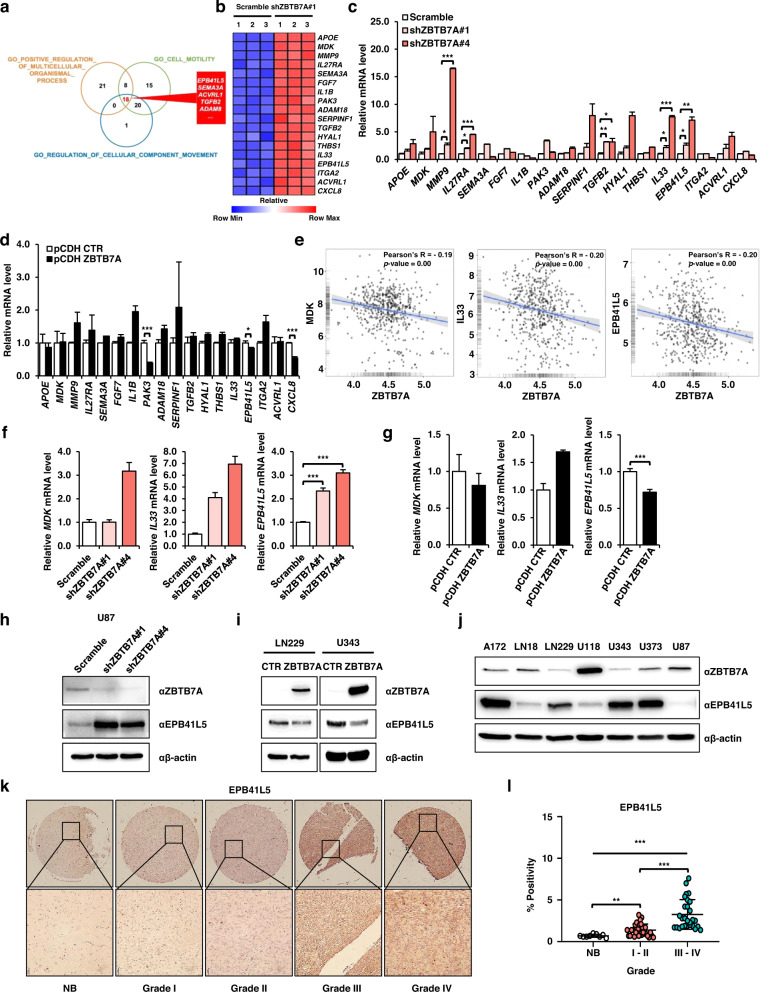
ZBTB7A negatively regulates EPB41L5. **a** The Venn diagram shows an overlap between GSEA results. **b** Heatmap of the expression and enrichment of 18 overlapping genes in the GSEA results. **c**, **d** mRNA expression levels of 18 overlapping genes in shZBTB7A U87 cells and pCDH ZBTB7A U343 cells. **e** Negative correlation of the gene expression levels of *MDK*, *IL33*, and *EPB41L5* with *ZBTB7A* (GlioVis). **f**, **g** mRNA expression levels of *MDK*, *IL33*, and *EPB41L5* in shZBTB7A U87 cells and pCDH ZBTB7A U343 cells. **h**, **i** Western blot analysis of ZBTB7A and EPB41L5 in shZBTB7A U87 cells and pCDH ZBTB7A LN229 and U343 cells. **j** Western blot analysis of ZBTB7A and EPB41L5 in GBM cell lines. **k** EPB41L5 IHC staining in increasing grades of glioma using TMA blots. **l** IHC analysis of EPB41L5 expression in TMA blots. NB, *n* = 9; Grades I–II, *n* = 30; III–IV, *n* = 28. Scale bars: 50 μm. *p < 0.05, **p < 0.01, ***p < 0.001.

Next, we analyzed the genes related to these three processes in ZBTB7A knockdown GBM cells. The upregulated genes overlapping with ZBTB7A were knocked down in three processes, and 18 genes were upregulated in all processes (Fig. 3a). The expression of 18 overlapping genes was confirmed using a heatmap (Fig. 3b). Therefore, 18 genes were upregulated when ZBTB7A expression was low, suggesting that there is an inverse correlation between the expression of ZBTB7A and the 18 target genes. To confirm the mRNA sequencing data, the mRNA expression levels of 18 target genes were analyzed in ZBTB7A knockdown and overexpression cells using qPCR (Fig. 3c, d). In addition, correlation analysis was performed on the TCGA-GBM dataset to confirm that the 18 target genes obtained from our mRNA-seq data had a inverse correlation with ZBTB7A, even in patient samples. We confirmed the inverse correlation of MDK, IL33, and EPB41L5 with ZBTB7A for three of the 18 target genes (Fig. 3e, Supplementary Fig. 2a). Therefore, we examined whether the mRNA levels of *MDK*, *IL33*, and *EPB41L5* increased in ZBTB7A shRNA-expressing GBM cells. As shown in Fig. 3f, the mRNA expression of *MDK*, *IL33*, and *EPB41L5* was inversely correlated with that of ZBTB7A. In addition, the expression of *EPB41L5* mRNA was significantly decreased in ZBTB7A-overexpressing GBM cells but not *in MDK* and *IL33* cells (Fig. 3g). Thus, EPB41L5 expression is directly affected by ZBTB7A expression. In addition, we confirmed that ZBTB7A and EPB41L5 had an inverse correlation at the protein level based on analysis of ZBTB7A knockdown U87 cells and ZBTB7A-overexpressing LN229 and U343 cells, respectively (Fig. 3h, i, Supplementary Fig. 2b, c). Furthermore, the protein expression levels of ZBTB7A and EPB41L5 were inversely correlated in GBM cell lines (Fig. 3j). The expression of EPB41L5 in tissues of patients with GBM (based on GBM TMAs) was observed using IHC staining, and the expression of EPB41L5 increased in the high-grade GBM tissues (Fig. 3k, l). Overall, ZBTB7A controls GBM tumorigenesis by regulating EPB41L5 expression.

### ZBTB7A overexpression suppresses GBM tumorigenesis through the transcriptional inhibition of EPB41L5

**Fig. 4:**
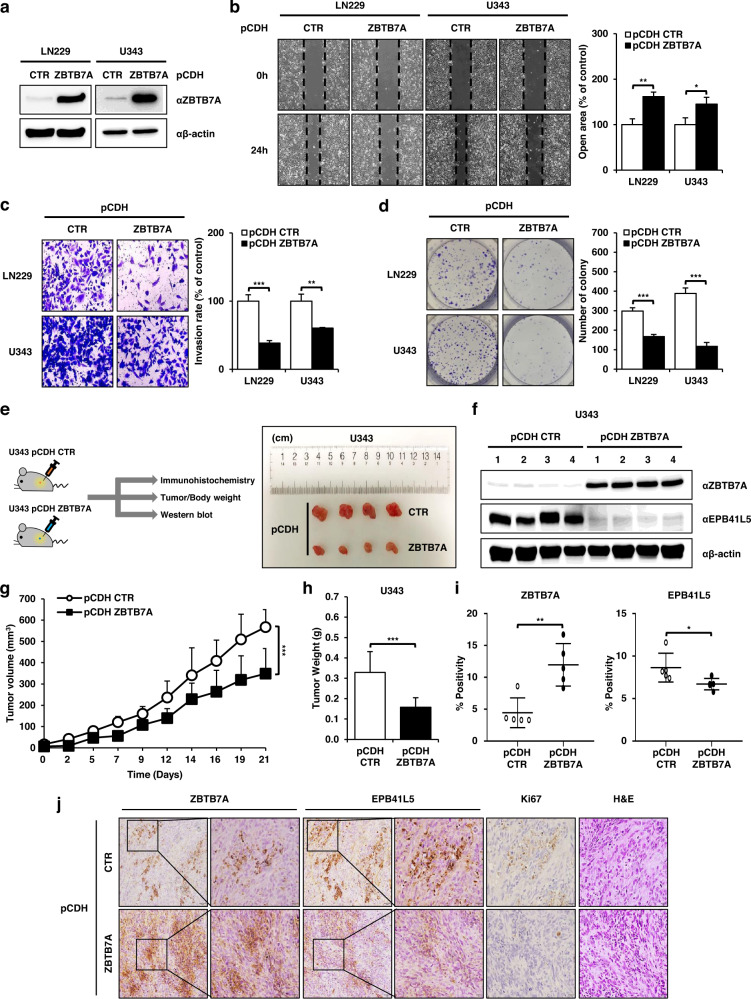
ZBTB7A expression regulates tumorigenesis in GBM cells. **a** Western blot analysis of ZBTB7A expression in pCDH ZBTB7A LN229 and U343 cells. **b** Migration assay of pCDH ZBTB7A LN229 and U343 cells after 24 h. **c** Transwell invasion assay of pCDH ZBTB7A LN229 and U343 cells after 48 h. **d** Effect of ZBTB7A overexpression on colony formation in pCDH ZBTB7A LN229 and U343 cells. **e** Schematic diagram and photograph of a subcutaneous tumor experiment using nude mice. **f** Western blot analysis of ZBTB7A and EPB41L5 in pCDH ZBTB7A U343 subcutaneous tumors. **g** Tumor volume was calculated according to the formula at 2–3 d intervals for 5 wk (*n* = 5 mice/group). **h** The weight of each tumor was measured (*n* = 8 mice/group). **i** IHC analysis of pCDH ZBTB7A U343 subcutaneous tumors using an IHC profiler (*n* = 5 mice/group). **j** IHC staining using anti-ZBTB7A, anti-EPB41L5, and anti-Ki67 antibodies and H&E staining. Scale bars: 50 μm, 20 μm. *p < 0.05, **p < 0.01, ***p < 0.001.

To confirm the previous results, we expressed ZBTB7A in LN229 and U343 cells, which have relatively low expression levels of ZBTB7A. The protein and mRNA expression levels of ZBTB7A were assessed in ZBTB7A-expressing LN229 and U343 cells (Fig. 4a, Supplementary Fig. 3a; cells with overexpression: pCDH-ZBTB7A and control cells: pCDH-CTR). To analyze the biological function of ZBTB7A in GBM cells, we performed various cell-based assays, such as cell migration, invasion, and colony-forming assays, in ZBTB7A-overexpressing LN229 and U343 cells. As shown in Fig. 4b, cell migration was decreased in ZBTB7A-overexpressing GBM cells compared to that in control cells. The invasiveness and colony formation ability of GBM cells were significantly decreased in ZBTB7A-overexpressing GBM cells compared to control cells (Fig. 4c, d), indicating that ZBTB7A inhibits tumorigenesis in GBM cells.

To confirm that ZBTB7A had the same inhibitory effect on GBM cell migration, invasion, and colonization, control cells (pCDH-CTR) and ZBTB7A-expressing U343 cells (pCDH- ZBTB7A) were injected subcutaneously into nude mice, and the tumor weight was monitored every 2 days for 6 weeks. As expected, tumor growth on ZBTB7A-expressing U343 cells was further reduced compared to that in control cells (Fig. 4e, Supplementary Fig. 3b). In addition, we confirmed whether the protein expression of ZBTB7A was normal in the ZBTB7A-expressing tumor using Western blotting (Fig. 4f) and whether the tumor volume and weights were suppressed in ZBTB7A-expressing U343 cells compared to the control cells (Fig. 4g, h). The survival rate was also higher in ZBTB7A-expressing U343 cells than in the control group (Supplementary Fig. 3c).

We analyzed the target genes of ZBTB7A in ZBTB7A-knockdown cancer cells using the ChIP-Atlas database and predicted that the target gene binds to suppress tumorigenesis as a transcription factor. We found that EPB41L5, a protein essential for cell migration, was involved in EMT (Supplementary Fig. 3d). In a previous study, we reported that the expression of EPB41L5 induced by TGF-β1 promotes the migration and invasion of gastric cancer cells via TGF-β signaling^34^. Therefore, we conducted the following experiment to determine whether ZBTB7A, a transcription factor, regulates transcriptional activity by targeting EPB41L5 in GBM cells. The protein expression of EPB41L5 and ZBTB7A was observed in mouse tumor tissues injected with control U343 cells and ZBTB7A-expressing U343 cells using IHC analysis. The expression of EPB41L5 was significantly decreased in ZBTB7A-overexpressing tumor tissues (Fig. 4i, j). Based on these results, we suggest that ZBTB7A acts as a transcriptional repressor that inhibits tumorigenesis by targeting EPB41L5 in GBM.

### ZBTB7A binds to the *EPB41L5* promoter to repress transcriptional activity

**Fig. 5:**
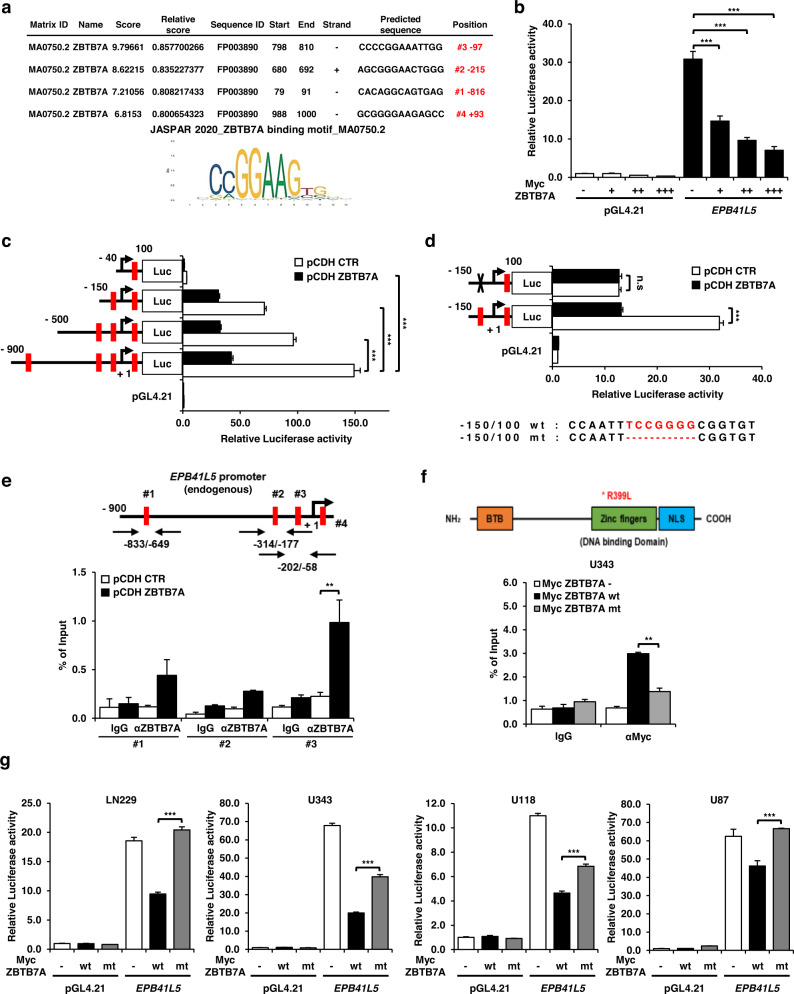
The ZBTB7A protein specifically binds to repress the transcriptional activity of the *EPB41L5* promoter. **a** Schematic of the 1 kb *EPB41L5* gene promoter and the ZBTB7A binding element within the ZBTB7A binding motif of JASPAR. **b** ZBTB7A suppresses *EPB41L5* promoter activity, as shown by the luciferase assay. **c** Repression by ZBTB7A in the *EPB41L5* promoter required a binding motif of ZBTB7A, as shown by a luciferase assay. The left panel shows the relative cleavage of the *EPB41L5* promoter. **d** Of the four potential ZBTB7A binding sites of the *EPB41L5* promoter, the most important (theZBTB7A#3 position) was deleted. Transcriptional activity of the depleted promoter was determined via a luciferase assay. **e** The upper panel shows the region amplified using ChIP primers in the *EPB41L5* promoter. The lower panel shows the amount of DNA precipitated using anti-ZBTB7A or IgG. **f** The domain structure of the ZBTB7A protein and the design of the ZBTB7A zinc finger mutant R399L. Then, Myc ZBTB7A wt and mt were transfected into U343 cells to show the amount of DNA precipitated by Myc antibody or IgG, and the -202/-58 region was amplified with a ChIP primer. **g** To determine the ability of ZBTB7A R399L to repress the activity of the *EPB41L5* promoter, we performed luciferase assays in LN229, U343, U118, and U87 cells. n.s; not significant, **p < 0.01, ***p < 0.001.

To investigate whether ZBTB7A and EPB41L5 are inversely correlated, we performed a promoter assay to confirm that ZBTB7A binds to the promoter of EPB41L5 as a transcription factor and negatively regulates its transcriptional activity. The binding motif sequence of ZBTB7A was confirmed using the JASPAR database, and the region where ZBTB7A binds was predicted in the 1 kb region (−900 to 100) of the *EPB41L5* promoter through the EPD database (Supplementary Fig. 4a, b). We also obtained overlapping results from the JASPAR and EPD databases for four regions that were significant for ZBTB7A binding in the *EPB41L5* promoter (Fig. 5a). In fact, the 1 kb region of the *EPB41L5* promoter was cloned into the pGL4.21 vector to perform the luciferase assay, and the ZBTB7A ORF was cloned into the Myc-tagged pSG5 vector for cotransfection into U343 cells with relatively low ZBTB7A expression. ZBTB7A was transfected in a dose-dependent manner, and we confirmed a gradual decrease in the transcriptional activity of EPB41L5 (Fig. 5b). To analyze transcriptional activity, the 1 kb full-length *EPB41L5* (−900 to 100: #1, −500 to 100: #2, −150 to 100: #3, and −40 to 100: #4) promoter regions were truncated. Furthermore, we measured transcriptional activity through the transfection of *EPB41L5* promoter-deleted mutants (*EPB41L5* promoter truncated constructs) in ZBTB7A-overexpressing U343 cells or control U343 cells. The transcriptional activity of *EPB41L5* promoter wild-type/deletion mutants was significantly reduced in ZBTB7A-overexpressing U343 cells compared to the control group, and when the −150 to 100 promoter regions (#3) were truncated, the transcriptional activity was drastically reduced in the ZBTB7A-overexpressing U343 cells and control cells (Fig. 5c). Based on these results, we confirmed that the promoter region, including the *EPB41L5* #3 (−150 to 100) position, was a significant ZBTB7A binding region.

To further confirm whether the *EPB41L5* #3 (−150 to 100) position is the ZBTB7A binding region, the *EPB41L5* promoter construct (#3-WT) or the specific binding sequence deleted form of the *EPB41L5* promoter construct (#3-MT) was transfected into ZBTB7A-overexpressing U343 cells or control cells, and promoter activity was assessed using a luciferase assay. The transcriptional activity of the ZBTB7A binding sequence deleted in the *EPB41L5* promoter (#3-MT) was not significantly different between ZBTB7A-expressing U343 cells and control cells compared with the wild-type *EPB41L5* promoter (#3-WT) (Fig. 5d). Therefore, we assessed whether ZBTB7A inhibits transcription by binding to region #3 of the *EPB41L5* promoter using a ChIP assay. Significantly more ZBTB7A was recruited to the #3 region of the *EPB41L5* promoter in ZBTB7A-expressing U343 cells than in control cells (Fig. 5e). In addition, we investigated whether R399L binds to the *EPB41L5* promoter region through site-directed mutagenesis of R399L in the zinc finger domain of ZBTB7A. ChIP assays were performed in U343 cells transfected with the empty control vector, wild-type (wt) Myc-ZBTB7A, and mutant-type (mt) Myc-ZBTB7A. mtZBTB7AR399L failed to bind to position #3 of the *EPB41L5* promoter (Fig. 5f). We also confirmed that mtZBTB7AR399L failed to repress the transcriptional activity of EPB41L5. We examined the transcriptional activity in the GBM cell lines LN229, U343, U118, and U87 transfected with the empty vector, wtZBTB7A, and mtZBTB7A (Fig. 5g). As expected, mtZBTB7AR399L did not inhibit the transcriptional repression of EPB41L5 compared to wtZBTB7A, and the wt/mt Myc-ZBTB7A protein was normally expressed in all cells. (Supplementary Fig. 4c). Thus, we suggest that ZBTB7A negatively regulates the transcriptional activity of EPB41L5 by binding to the ZBTB7A #3 position of the *EPB41L5* promoter.

### ZBTB7A knockdown increases GBM tumorigenesis through the transcriptional activation of EPB41L5

**Fig. 6:**
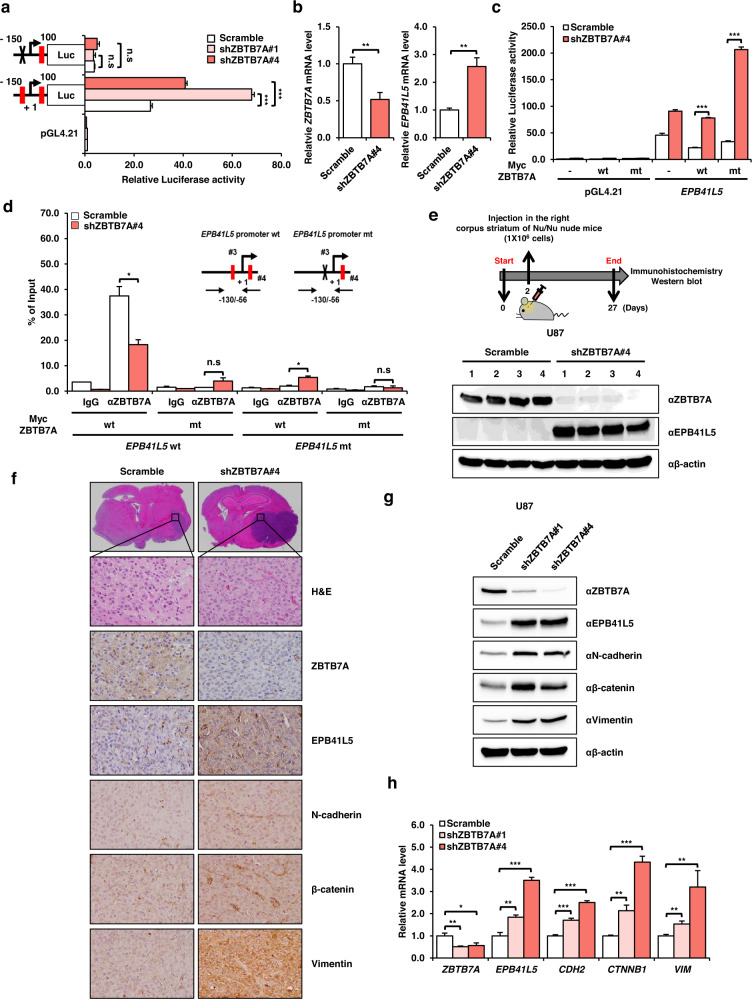
EPB41L5 induces EMT and increases tumorigenesis. **a** The transcriptional activity of the *EPB41L5* promoter with deletion of the #3 position in shZBTB7A U87 cells was investigated by luciferase assay. **b** mRNA levels of *ZBTB7A* and *EPB41L5* in shZBTB7A#4 U87 cells. **c** To measure the ability of ZBTB7A R399L to repress promoter activity when ZBTB7A protein expression was low, we performed a luciferase assay in shZBTB7A#4 U87 cells. **d** The −130/−56 region was amplified with ChIP primers, showing the amount of DNA precipitation by ZBTB7A antibody or IgG by cotransfection of Myc ZBTB7A and *EPB41L5* promoters in shZBTB7A#4 U87 cells. **e** Schematic of the experimental procedure of orthotopic GBM nude mouse models injected with shZBTB7A#4 U87 cells and Western blot analysis of ZBTB7A and EPB41L5 in tumor samples (*n* = 4 mice/group). **f** H&E staining and IHC staining of anti-ZBTB7A, EPB41L5, and EMT markers (N-cadherin, β-catenin, and vimentin) in orthotopic GBM nude mouse models injected with shZBTB7A#4 U87 cells. **g**, **h** Expression of EMT markers by Western blot and qPCR analyses in shZBTB7A U87 cells. Scale bars: 20 μm. n.s; not significant, *p < 0.05, **p < 0.01, ***p < 0.001.

Next, we performed a luciferase assay to determine whether transcriptional activation of EPB41L5 was increased by ZBTB7A knockdown. The transcriptional activation of EPB41L5 was greatly increased in the −150 to 100 region (#3) of the *EPB41L5* promoter but not in the #3 mutant promoter after ZBTB7A knockdown (Fig. 6a). In addition, the mRNA expression of *EPB41L5* and *ZBTB7A* was confirmed in shZBTB7A-expressing U87 cells, and the transcriptional activity of EPB41L5 was measured in shZBTB7A-expressing U87 cells that target the 3′UTR *EPB41L5* promoter. mtZBTB7A expression greatly induced the transcriptional activity of EPB41L5. The activities of wtZBTB7A and the control were similar (Fig. 6b, c, Supplementary Fig. 4d). In addition, a ChIP assay was performed in shZBTB7A-expressing U87 cells cotransfected with the EPB41L5 promoter and wtZBTB7A or mtZBTB7A.

In the previous experiments, mtZBTB7A failed to bind the *EPB41L5* promoter, and wtZBTB7A did not bind the #3 mutation region of the *EPB41L5* promoter (Fig. 6d). Therefore, we monitored the development and progression of tumors derived from shZBTB7A-expressing GBM cells in an orthotopic xenograft mouse model. Control U87 and shZBTB7A knockdown U87 cells were injected into the right corpus striatum of the mouse brain. After observation for 4 weeks, the GBM tumor volume was significantly increased in a mouse injected with shZBTB7A-expressing U87 cells compared to that in the control mice, and the protein expression levels of ZBTB7A and EPB41L5 in GBM tumor tissues were inversely correlated (Fig. 6e).

We observed the expression of EMT markers (N-cadherin, β-catenin, and vimentin) and EPB41L5 (Fig. 6f). Similarly, the protein and mRNA expression of N-cadherin, β-catenin, and vimentin was confirmed in shZBTB7A U87 cells, and both the protein and mRNA levels were increased in these cells versus control cells (Fig. 6g, h). Taken together, our results show that the knockdown of ZBTB7A induces transcriptional activation of EPB41L5 and promotes GBM tumorigenesis via the EMT process.

## Discussion

GBM is a highly mobile, invasive, and lethal primary tumor that is resistant to all therapies and has a poor prognosis^35^. Although the frequent occurrence of genetic abnormalities has been reported in various cohort profiling studies, survival is difficult to predict. This result implies that there are other factors influencing abnormal gene expression. ZBTB7A, a transcriptional activator or repressor known to play an important role in tumorigenesis and metastasis in various human cancers, is downregulated in GBM^25^. ZBTB7A is required for the regulation of tumor growth in cancers, including GBM. However, it is not clear how ZBTB7A functions as a transcriptional activator or repressor of tumor growth in GBM cells. In our study, ZBTB7A provided insight into the mechanism of a tumor suppressor in GBM, and its deficiency significantly increased the tumor growth of GBM. In particular, among the various targets regulated by ZBTB7A, the *EPB41L5* gene, which is involved in cell migration and the EMT process, was discovered through ChIP-Atlas and mRNA-seq analyses. In a previous study, we reported that EPB41L5 was induced by TGF-β signaling in gastric cancer and increased cell mobility and invasiveness and the expression of various EMT markers^34^. Therefore, because the expression of ZBTB7A is low in GBM, we confirmed that GBM growth is regulated through overexpression of ZBTB7A. ZBTB7A inhibited the migration, invasion, and proliferation of GBM cells. However, the growth of GBM tumors was promoted by ZBTB7A knockdown, and GBM tumor volume was significantly increased in the in vivo mouse GBM model. In addition, we subcutaneously injected ZBTB7A-overexpressing U343 cells into nude mice. ZBTB7A overexpression did not promote tumor growth in vivo in GBM models, but it suppressed the expression of EPB41L5, a target of ZBTB7A. Furthermore, shZBTB7A-expressing U87 cells enhanced GBM tumorigenesis in an in vivo orthotopic xenograft mouse model of GBM. To clarify our findings, mRNA sequencing analysis was performed to confirm the mechanism by which ZBTB7A, a transcriptional repressor, inhibits tumorigenesis by targeting EPB41L5 in GBM. When ZBTB7A was depleted in GBM cells, tumor migration and EMT-related gene expression were significantly increased. In this process, we verified once again that EPB41L5 is an important target and confirmed that the survival rate of patients with GBM decreased with increasing EPB41L5 expression.

To demonstrate the transcriptional regulation of EPB41L5 by ZBTB7A as a transcriptional repressor, we predicted that the promoter of EPB41L5 had a ZBTB7A motif and found that ZBTB7A negatively regulated the transcription level of EPB41L5 by binding directly to −97 bp (*EPB41L5* #3 position) close to the EPB41L5 transcription start site. In addition, we clearly showed that the ZBTB7A-EPB41L5 axis could play a central role in promoting tumor growth in GBM by cooperatively promoting the EMT process. However, while we have demonstrated that the ZBTB7A-EPB41L5 axis significantly promotes GBM tumor growth, we do not yet understand how this axis is activated depending on the cellular microenvironment. Therefore, we considered the correlation between tumorigenesis and the tumor microenvironment essential for the growth of GBM cells through epigenetic regulation of the target gene with ZBTB7A.

Chromatin modification by the epigenome is important in determining the transcriptional activation of EPB41L5 by ZBTB7A, and ZBTB7A regulates the expression of target genes by recruiting epigenetic modifiers of corepressors, such as BCoR, NCoRs, HDACs, SIN3A, and MBD3^36–38^. Based on the report that ZBTB7A recruits various corepressors, we suggest that ZBTB7A is a central corepressor complex and has the potential to regulate transcription by controlling chromatin remodeling in the EPB41L5 promoter. In addition, GBM tumorigenesis is determined by the secretomes of the tumor microenvironment through epigenetic regulation of corepressors and coactivators by ZBTB7A. As a result, ZBTB7A-mediated EPB41L5 inhibition should be considered as a therapeutic strategy for GBM.

## Supplementary information

Supplementary Information

## Data Availability

The raw mRNA-seq data reported in this document have been deposited into the GEO under accession number GSE196320, available at.
