# Supplementary material for: ZBTB7A suppresses glioblastoma tumorigenesis through the transcriptional repression of EPB41L5
Source: Exp Mol Med. 2023 Jan 4;55(1):43–54. doi: 10.1038/s12276-022-00908-8 (PMC9898510; doi:10.1038/s12276-022-00908-8)
Supplement: Supplementary file 1 — Supplementary Information [file 12276_2022_908_MOESM1_ESM.pdf]

Supplementary Fig. 1

a

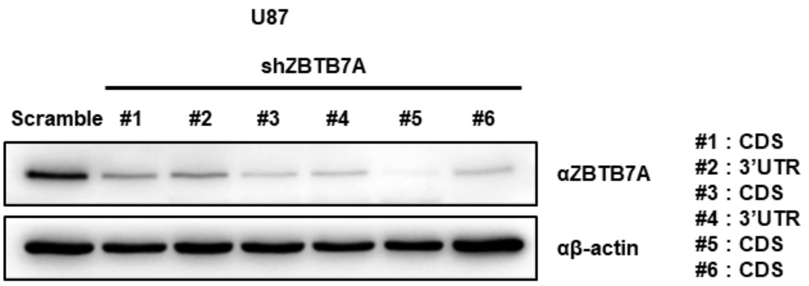

b

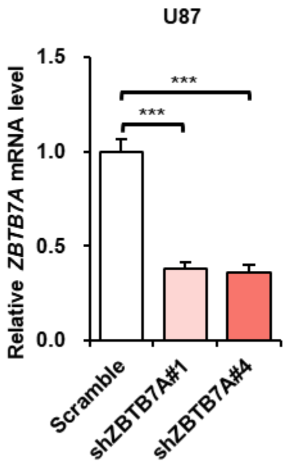

Supplementary Fig. 2

a

Gene Symbol	Fold change	estimate	statistic	p.value	parameter	conf.low	conf.high	method	alternative
**APOE**	42.780	-0.04	-0.91	0.37	536.00	-0.12	0.05	Pearson's product-moment correlation	two.sided
**MDK**	5.319	-0.19	-4.58	0.00	536.00	-0.27	-0.11	Pearson's product-moment correlation	two.sided
**MMP9**	4.310	-0.05	-1.16	0.25	536.00	-0.13	0.03	Pearson's product-moment correlation	two.sided
**IL27RA**	3.537	0.11	2.67	0.01	536.00	0.03	0.2	Pearson's product-moment correlation	two.sided
**SEMA3A**	3.023	0.02	0.53	0.60	536.00	-0.06	0.11	Pearson's product-moment correlation	two.sided
**FGF7**	2.701	0.02	0.53	0.60	536.00	-0.06	0.11	Pearson's product-moment correlation	two.sided
**IL1B**	2.612	0.06	1.43	0.15	536.00	-0.02	0.15	Pearson's product-moment correlation	two.sided
**PAK3**	2.609	0.10	2.38	0.02	536.00	0.02	0.19	Pearson's product-moment correlation	two.sided
**ADAM8**	2.597	0.17	3.88	0.00	536.00	0.08	0.25	Pearson's product-moment correlation	two.sided
**SERPINF1**	2.593	0.01	0.28	0.78	536.00	-0.07	0.1	Pearson's product-moment correlation	two.sided
**TGFB2**	2.480	0.03	0.80	0.43	536.00	-0.05	0.12	Pearson's product-moment correlation	two.sided
**HYAL1**	2.243	0.12	2.89	0.00	536.00	0.04	0.21	Pearson's product-moment correlation	two.sided
**THBS1**	2.114	0.06	1.40	0.16	536.00	-0.02	0.14	Pearson's product-moment correlation	two.sided
**IL33**	2.111	-0.20	-4.63	0.00	536.00	-0.28	-0.11	Pearson's product-moment correlation	two.sided
**EPB41L5**	2.082	-0.20	-4.67	0.00	536.00	-0.28	-0.11	Pearson's product-moment correlation	two.sided
**ITGA2**	2.077	0.14	3.39	0.00	536.00	0.06	0.23	Pearson's product-moment correlation	two.sided
**ACVRL1**	2.027	0.09	2.11	0.04	536.00	0.01	0.17	Pearson's product-moment correlation	two.sided
**CXCL8**	Gene not available for this dataset								

b

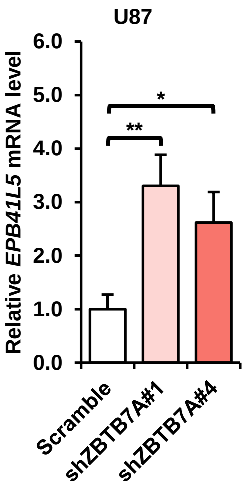

c

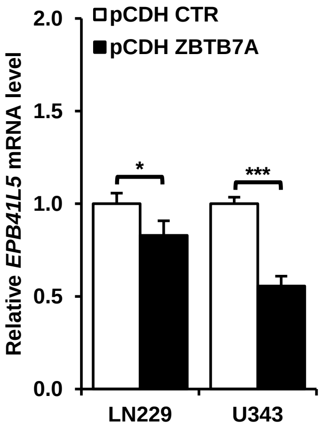

Supplementary Fig. 3

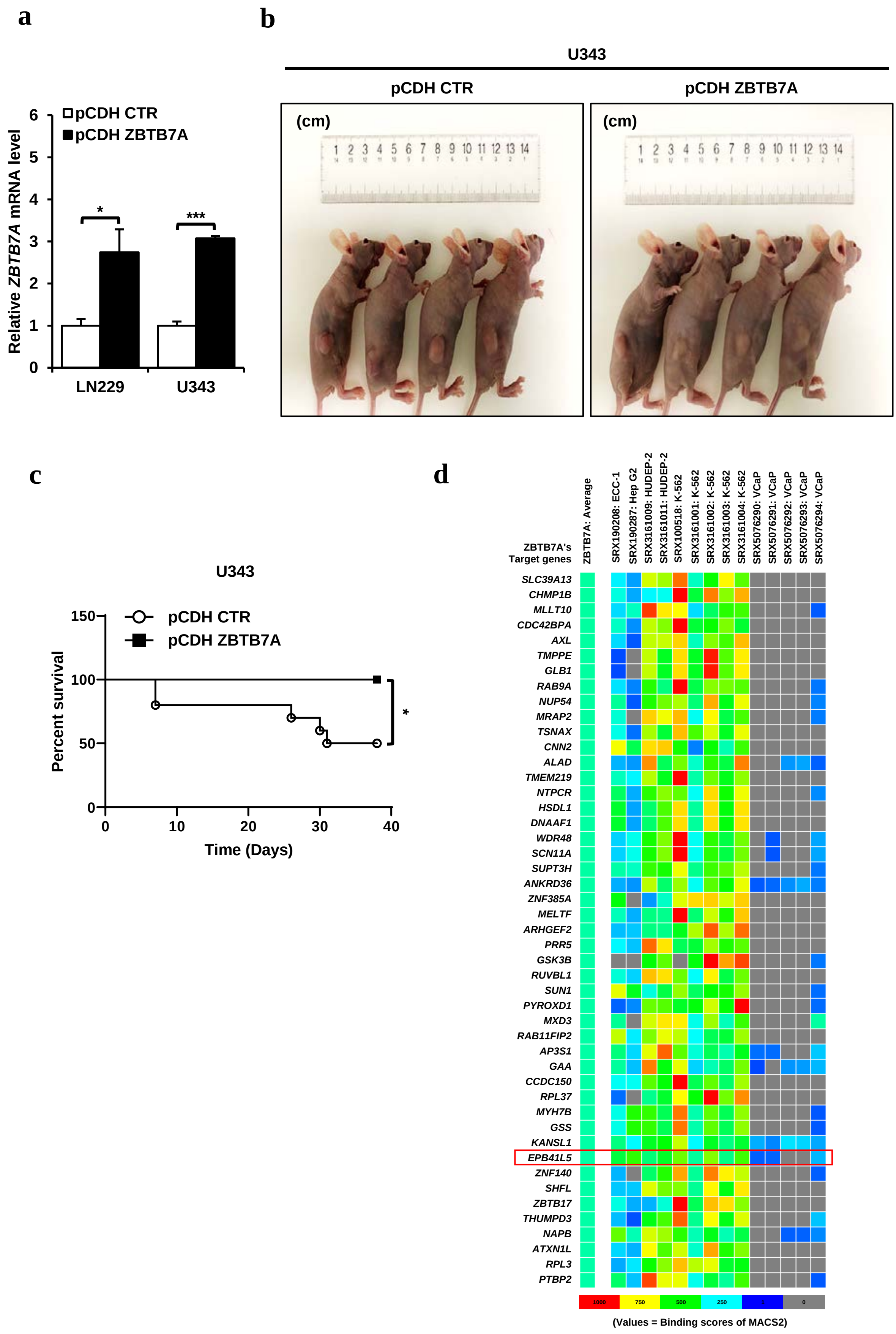

Supplementary Fig. 4

a

Library : Transcription Factor Motifs (JASPAR CORE 2018 vertebrates) Motif : ZBTB7A
From : -1000 To : 100 bp relative to TSS and a cut-off (*p*-value) of : 0.001

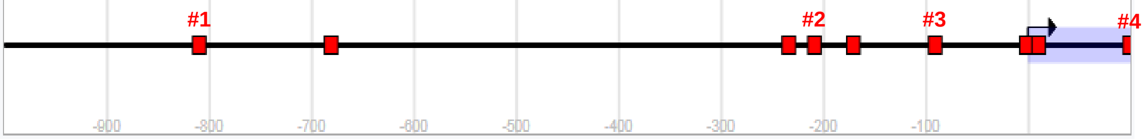

ZBTB7A [*p*-value = 0.001] : -816, -687, -240, -215, -177, -97, -8, -4, 93

Library : Transcription Factor Motifs (JASPAR CORE 2018 vertebrates) Motif : ZBTB7A
From : -1000 To : 100 bp relative to TSS and a cut-off (*p*-value) of : 0.0001

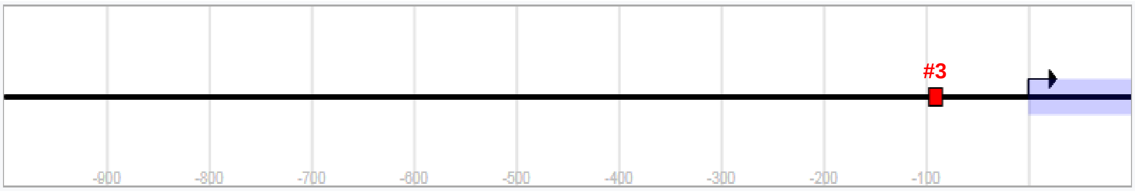

ZBTB7A [*p*-value = 0.0001] : -97

b

>FP003890 EPB41L5_1 :+U EU:NC; range -900 to 100.

TGGGATTACAGGAGTGAGCCACCGCGCCCAGCCAAATGCCACCTTCTTAT
TGTGACCTGCTATGTAAAATCGCAAACCCTCACTGCCTGTGGTTATTCCC #1
TACTTGTACTTTTCTTTTTAGCATTTACCAATGTCTGACAGTATTTAATT
TCACTAATTTATCTCGTTTATTTTATGTCTCGTTTATATTCCTGCTATAA
CGTAAACTCCGGGAAGGATTTTTGTCTGTCTTGGTTGTCTGTCGTATCTC
GTGTCTAGAACACAGAACTCACAGTAAGTAGGCACTTCAATCAATTACCT
GTAGAATGAACGGACTGAATGTCACCTTGTTAGAGTACCCTCCTGACCAC
ACTCTGCTAAGCAGTAATCTTTTATTTCTCTTTCCCAGTTTTATTTTTCC
TCATTTACACTTAATCACCTTGTGACACCTATTTGTTTACTGTTTCCCCA
GTAGAGTGAAAGTCACTTTAACAGCAGAGATTTAAATGTATTTTAATCCC
TACAAAGGAGAAAAAGGGAGGAAATACGTGACAGGCTCTCAATAAGTATT
TGCGAAGGATTTAATCTCTTGAACGAATGGATGAATGAATCTAGCAGCCA
GCTCCACCAGCCTCCTTCGTGATAGGACTAACTGGGGTCTGTCTGCGGAC
GGGCGCCTCCTCCGCGGGCGGAGGGCCGCAGCGGGAACTGGGTTTCGGCA #2
GCGCCCCTTTAAACCAGCCGCGGACGCCCGGCTGCTGGCGCTAGTTCCAG
CCCGGCCGGGCCCGGCCTCGCCGGTTTCTCTCCAGTCGCCGCGCCGGCCA #3
ATTTCCGGGGCGGTGTCATCGCCCGTTTAAGAGCGGAGCGCTCCGCCCTG
GGGGCGGAGCTGGGAGGGAGCTTTAAGGGGTGGACGGGCGGGAGGTCGGG
GTCCTCCGGGGATTAGAGCCGGTGGGCTCGTTGTGGGCGCCATTTCTCGG
CGTCTACCGAGGAGCCGCCCCTTTCTCAGCCTTGCTCGGCTCTTCCCCGC #4

c

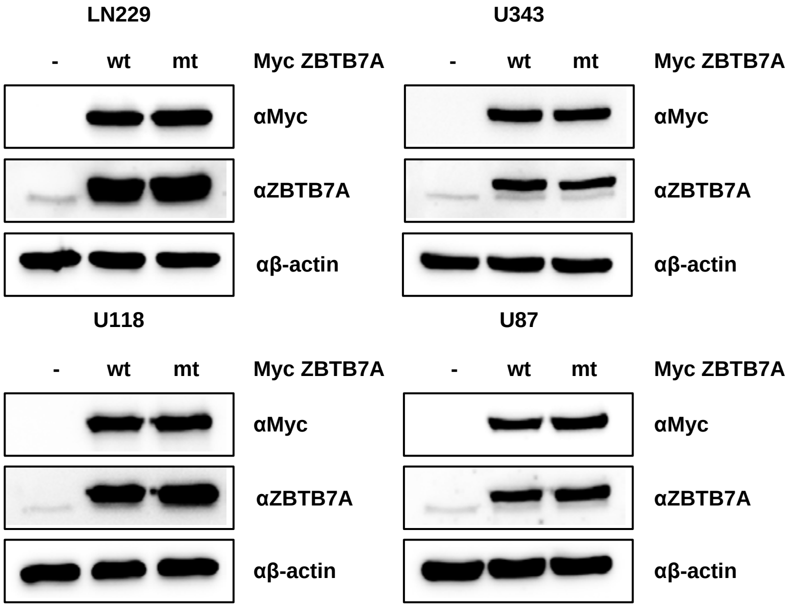
LN229
U343
U118
U87
- wt mt Myc ZBTB7A
αMyc
αZBTB7A
αβ-actin

d

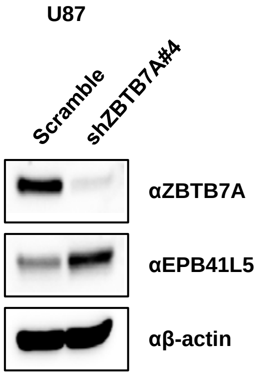
U87
Scramble
shZBTB7A#4
αZBTB7A
αEPB41L5
αβ-actin

Supplementary Figure Legends

Supplementary Fig. 1. Expression of ZBTB7A knockdown U87 cells. **a, b** Western blot and qPCR analysis of ZBTB7A silencing in U87 cells. ***$p < 0.001$.

Supplementary Fig. 2. Reverse correlation targets with ZBTB7A in patient samples. a Correlation of 18 overlapping genes with ZBTB7A in the TCGA-GBM dataset (GlioVis). **b, c** *EPB41L5* mRNA level in shZBTB7A U87 cells and pCDH ZBTB7A LN229, U343 cells. *$p < 0.05$, **$p < 0.01$, ***$p < 0.001$

Supplementary Fig. 3. Reduction of subcutaneous tumor by ZBTB7A overexpression. a mRNA level of *ZBTB7A* in pCDH ZBTB7A LN229, U343 cells. **b** Tumor size confirmed 5 weeks after subcutaneous injection of pCDH ZBTB7A U343 cells (n = 4 mice/group). **c** The survival rate of a mouse model injected subcutaneously with pCDH ZBTB7A U343 cells. **d** Target gene and MACS2 binding score of potential ZBTB7A at ± 1 kb distance using ChIP-Atlas. *$p < 0.05$, ***$p < 0.001$

Supplementary Fig. 4. Schematic diagram of the binding site of ZBTB7A in the *EPB41L5* promoter region. a Predicted binding site to which the ZBTB7A motif can bind in the *EPB41L5* promoter region (-900 to 100) (EPD). **b** *EPB41L5* promoter sequence from -900 to 100 region (EPD). **c** Protein levels of Myc ZBTB7A wt and mt in LN229, U343, U118 and U87 cells. **d** Western blot analysis of shZBTB7A#4 U87 cells.

Supplementary Table 1. List of primer for shRNA, qPCR, ChIP

**shRNA**			
shZBTB7A#1	CDS	Sigma-Aldrich	TRCN0000137332
shZBTB7A#2	3’UTR	Sigma-Aldrich	TRCN0000136851
shZBTB7A#3	CDS	Sigma-Aldrich	TRCN0000137998
shZBTB7A#4	3’UTR	Sigma-Aldrich	TRCN0000138671
shZBTB7A#5	CDS	Sigma-Aldrich	TRCN0000138519
shZBTB7A#6	CDS	Sigma-Aldrich	TRCN0000138716

**qPCR**	
ZBTB7A	F : CATCTGCGAGAAGGTCATCCA<br>R : AGGTCGTAGTTGTGGGCAAAG
EPB41L5	F : GAAAGAAGGCCCAGCAAACG<br>R : AGATCTCATCCCCCAAGCCT
APOE	F : GTTGCTGGTCACATTCCTGG<br>R : GCAGGTAATCCCAAAAGCGAC
MDK	F : CGCGGTCGCCAAAAAGAAAG<br>R : TACTTGCAGTCGGCTCCAAAC
MMP9	F : TGTACCGCTATGGTTACACTCG<br>R : GGCAGGGACAGTTGCTTCT
IL27RA	F : GACCCTTGGGCGACTTGAA<br>R : TCTGGAGGTGTAACTCGGAGG
SEMA3A	F : GTGCCAAGGCTGAAATTATCCT<br>R : CCCACTTGCATTCATCTCTTCT
FGF7	F : TCCTGCCAACTTTGCTCTACA<br>R : CAGGGCTGGAACAGTTCACAT
IL1B	F : ATGATGGCTTATTACAGTGGCAA<br>R : GTCGGAGATTCGTAGCTGGA
PAK3	F : CCAGGCTTCGCTCTATCTTCC<br>R : TCAAACCCCACATGAATCGTATG
ADAM18	F : GTCACAGTTCCACGGAAGATT<br>R : CAGCATATCCTTGGTAATGGCA

SERPINF1	F : TTCAAAGTCCCCGTGAACAAG<br>R : GAGAGCCCGGTGAATGATGG
TGFB2	F : CAGCACACTCGATATGGACCA<br>R : CCTCGGGCTCAGGATAGTCT
HYAL1	F : CGATATGGCCCAAGGCTTTAG<br>R : ACCACATCGAAGACACTGACAT
THBS1	F : AGACTCCGCATCGCAAAGG<br>R : TCACCACGTTGTTGTCAAGGG
IL33	F : GTGACGGTGTTGATGGTAAGAT<br>R : AGCTCCACAGAGTGTTCCTTG
ITGA2	F : CCTACAATGTTGGTCTCCCAGA<br>R : AGTAACCAGTTGCCTTTTGGATT
ACVRL1	F : CGAGGGATGAACAGTCCTGG<br>R : GTCATGTCTGAGGCGATGAAG
CXCL8	F : TTTTGCCAAGGAGTGCTAAAGA<br>R : AACCCTCTGCACCCAGTTTTC
CDH2	F : CCATCACTCGGCTTAATGGT<br>R : GATGATGATGCAGAGCAGGA
CTNNB1	F : AGCTTCCAGACACGCTATCAT<br>R : CGGTACAACGAGCTGTTTCTAC
VIM	F : CCCTCACCTGTGAAGTGGAT<br>R : TCCAGCAGCTTCCTGTAGGT

**ChIP**	
-833/-649	F : AATCGCAAACCCTCACTGCC<br>R : ACGAGATACGACAGACAACCA
-314/-177	F : GAATCTAGCAGCCAGCTCCA<br>R : TCCGCGGCTGGTTTAAAGG
-202/-58	F : CAGCGCCCCTTTAAACCAG<br>R : GAGCGCTCCGCTCTTAAAC

-130/-56	F : CCGGTTTCTCTCCAGTCGC<br>R : TAGACGCCGAGAAATGGCG

Supplementary Table 2. List of GSEA core enrichment genes

GO_POSITIVE_REGULATION_OF_MULTICELLULAR_ORGANISMAL_PROCESS

SYMBOL	RANK METRIC SCORE	RUNNING ES	CORE ENRICHMENT
APOE	4.771662712	0.01764514	Yes
MDK	3.417632103	0.020849245	Yes
MMP9	3.113207579	0.023975836	Yes
EBI3	3.048780441	0.03315349	Yes
IL27RA	2.796052694	0.036155492	Yes
DIO2	2.76886034	0.045346245	Yes
FST	2.739628077	0.05547712	Yes
CHI3L1	2.707902193	0.061297804	Yes
PTK7	2.697974205	0.071274646	Yes
FOXS1	2.602739811	0.07775467	Yes
SEMA3A	2.512274981	0.07865907	Yes
IDO1	2.435897589	0.08557034	Yes
CTNNBIP1	2.358490705	0.09009894	Yes
SLITRK6	2.351916552	0.09774788	Yes
FGF7	2.3039217	0.10417111	Yes
CLSTN3	2.297008514	0.11266523	Yes
PAK3	2.238806009	0.118847676	Yes
IL1B	2.230480433	0.12604755	Yes
ADAM8	2.219878197	0.13425644	Yes
G0S2	2.217725515	0.14245737	Yes
SERPINF1	2.209302425	0.14957894	Yes
TMEM100	2.144563913	0.1501718	Yes
LBH	2.138643026	0.1580803	Yes
TGFB2	2.127413273	0.16594726	Yes
RND2	2.116564512	0.17272589	Yes
C3	2.096502304	0.1783821	Yes
CRABP2	2.095182896	0.18612988	Yes
CSF2	1.917983294	0.17330626	Yes
HYAL1	1.899224758	0.17613652	Yes
PRL	1.861702085	0.17673162	Yes
IL18	1.827586174	0.17615232	Yes
THBS1	1.787878752	0.16808867	Yes

IL33	1.783216834	0.17468283	Yes
EPB41L5	1.752136707	0.17382453	Yes
ITGA2	1.750187993	0.17924833	Yes
AQP1	1.720000029	0.17931943	Yes
MYRF	1.633663297	0.16649263	Yes
CXCL8	1.625263095	0.17145446	Yes
GCH1	1.617647052	0.17429172	Yes
ACVRL1	1.601941705	0.1781191	Yes
S100A9	1.596900463	0.18297607	Yes
TNFSF12	1.584507108	0.18569076	Yes
SULF2	1.551724195	0.18199492	Yes
FOXG1	1.518171191	0.17398213	Yes
DISC1	1.512605071	0.17957559	Yes
PTPRJ	1.503567696	0.18094277	Yes
PLXND1	1.501650214	0.18649572	Yes

GO_CELL_MOTILITY

SYMBOL	RANK METRIC SCORE	RUNNING ES	CORE ENRICHMENT
APOE	4.771662712	0.01820441	Yes
RARRES2	3.725398302	0.0272251	Yes
MDK	3.417632103	0.037148472	Yes
MMP9	3.113207579	0.040718324	Yes
IL27RA	2.796052694	0.04100135	Yes
CXCL1	2.72684741	0.048289303	Yes
PTK7	2.697974205	0.05442868	Yes
CLDN4	2.579617977	0.059078094	Yes
SEMA3B	2.574967384	0.068901874	Yes
SEMA6B	2.556270123	0.07865432	Yes
SEMA3A	2.512274981	0.08408523	Yes
SEMA4G	2.487684727	0.09357602	Yes
PLAU	2.417070389	0.099682145	Yes
FGF7	2.3039217	0.101202905	Yes
PAK3	2.238806009	0.106628925	Yes
IL1B	2.230480433	0.11410003	Yes
ADAM8	2.219878197	0.12256911	Yes
SERPINF1	2.209302425	0.12892099	Yes
DOCK4	2.198443651	0.13626987	Yes
MCAM	2.154434919	0.141374	Yes
TGFB2	2.127413273	0.14637506	Yes
RND2	2.116564512	0.15341155	Yes

ELMO1	2.004608393	0.1485983	Yes
SCG2	1.911925435	0.14343144	Yes
IGFBP5	1.911764741	0.15072502	Yes
HYAL1	1.899224758	0.15693235	Yes
LAMA4	1.840369463	0.15356934	Yes
SH3BP1	1.83544302	0.16057175	Yes
IL24	1.814159274	0.16126244	Yes
CXCL3	1.802973986	0.1639873	Yes
ABI3	1.792452812	0.16874886	Yes
THBS1	1.787878752	0.17349297	Yes
IL33	1.783216834	0.18029614	Yes
SEMA6D	1.780821919	0.18709016	Yes
MTUS1	1.769025207	0.19176234	Yes
EPB41L5	1.752136707	0.19533166	Yes
COL1A2	1.751157761	0.20201252	Yes
ITGA2	1.750187993	0.20868967	Yes
FOXF1	1.741154552	0.21429396	Yes
CADM4	1.735751271	0.2198776	Yes
PODXL	1.700819612	0.22013588	Yes
MMP1	1.684881568	0.22448705	Yes
TNFSF14	1.640625	0.220362	Yes
CXCL8	1.625263095	0.22448571	Yes
FMNL1	1.620498657	0.23066808	Yes
GRB14	1.61728394	0.23372294	Yes
ACVRL1	1.601941705	0.2387961	Yes
DOCK8	1.59793818	0.2448924	Yes
S100A9	1.596900463	0.25098476	Yes
ASAP3	1.59685874	0.25707695	Yes
TNFSF12	1.584507108	0.26104516	Yes
FERMT3	1.526315808	0.24609978	Yes
FOXG1	1.518171191	0.24981493	Yes
DISC1	1.512605071	0.2555857	Yes
RHOF	1.509433866	0.2592675	Yes
PTPRJ	1.503567696	0.26396534	Yes
PLXND1	1.501650214	0.2696943	Yes
IFITM1	1.475253463	0.2628615	Yes
HGF	1.473128557	0.2674432	Yes
ADAMTS12	1.464542627	0.26887694	Yes
BST2	1.428571343	0.2701734	Yes

GO_REGULATION_OF_CELLULAR_COMPONENT_MOVEMENT

SYMBOL	RANK METRIC SCORE	RUNNING ES	CORE ENRICHMENT
APOE	4.771662712	0.023490772	Yes
RARRES2	3.725398302	0.036830813	Yes
MDK	3.417632103	0.050655726	Yes
MMP9	3.113207579	0.057981964	Yes
CNIH2	2.83258605	0.06392671	Yes
IL27RA	2.796052694	0.076691605	Yes
CLDN4	2.579617977	0.075391	Yes
SEMA3B	2.574967384	0.088067494	Yes
SEMA6B	2.556270123	0.10065195	Yes
SEMA3A	2.512274981	0.109019816	Yes
SEMA4G	2.487684727	0.12126662	Yes
PLAU	2.417070389	0.1301658	Yes
FGF7	2.3039217	0.13450794	Yes
PAK3	2.238806009	0.14252953	Yes
IL1B	2.230480433	0.15251012	Yes
ADAM8	2.219878197	0.16343853	Yes
SERPINF1	2.209302425	0.17231487	Yes
DOCK4	2.198443651	0.18213774	Yes
MCAM	2.154434919	0.18974398	Yes
TGFB2	2.127413273	0.19721718	Yes
IGFBP5	1.911764741	0.17862874	Yes
HYAL1	1.899224758	0.18697858	Yes
LAMA4	1.840369463	0.18603867	Yes
SH3BP1	1.83544302	0.1950745	Yes
IL24	1.814159274	0.19800556	Yes
ABI3	1.792452812	0.19982976	Yes
THBS1	1.787878752	0.20663144	Yes
IL33	1.783216834	0.21541017	Yes
SEMA6D	1.780821919	0.2241771	Yes
MTUS1	1.769025207	0.23088598	Yes
EPB41L5	1.752136707	0.2365117	Yes
ITGA2	1.750187993	0.24412782	Yes
FOXF1	1.741154552	0.25169948	Yes
CADM4	1.735751271	0.25924456	Yes
PODXL	1.700819612	0.26161763	Yes
TNFSF14	1.640625	0.2566944	Yes
CXCL8	1.625263095	0.26269552	Yes
ACVRL1	1.601941705	0.26458183	Yes
DOCK8	1.59793818	0.27244845	Yes
